# Supplementary material for: Expression Analysis of mRNA Decay of Maternal Genes during Bombyx mori Maternal-to-Zygotic Transition
Source: Int J Mol Sci. 2019 Nov 12;20(22):5651. doi: 10.3390/ijms20225651 (PMC6887711; doi:10.3390/ijms20225651)
Supplement: Supplementary file 1 [file ijms-20-05651-s001.zip › ijms-596242 supplementary/Figure S1.docx]

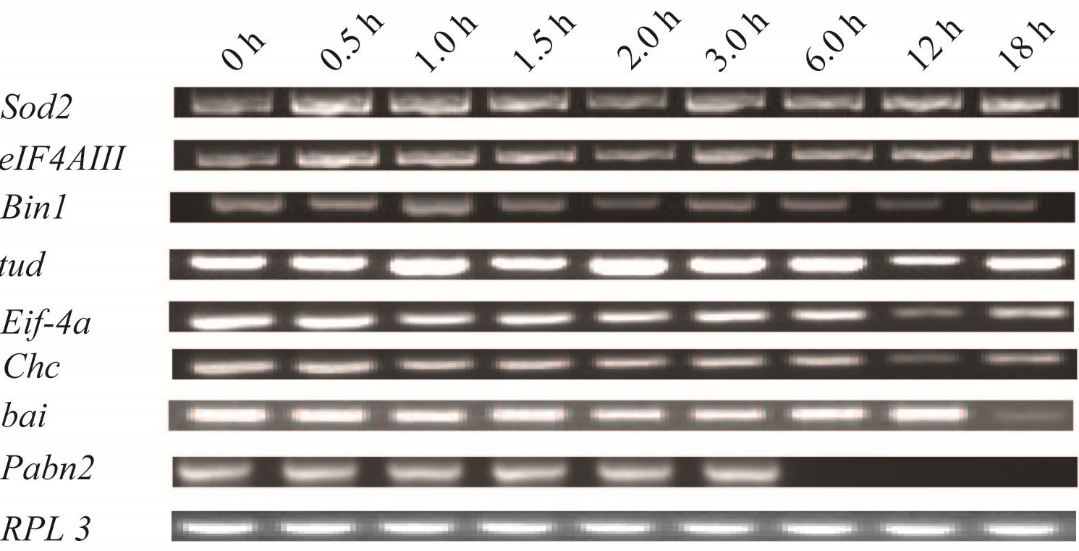


Figure S1. Cluster 1 maternal gene expression patterns in fertilized eggs from 0 to 18 h after spawning. Reverse transcription (RT)-PCR was performed and the *RPL3* gene was used as internal control.
